# Atypical Presentation of Microscopic Polyangiitis in an Elderly Woman

**DOI:** 10.7759/cureus.82778

**Published:** 2025-04-22

**Authors:** Chukwunonso B Ubanatu, Ogheneyoma Akpoviroro, Uwandu Queeneth

**Affiliations:** 1 Internal Medicine, Geisinger Wyoming Valley Medical Center, Wilkes-Barre, USA

**Keywords:** antineutrophil cytoplasmic autoantibody (anca), antinuclear antibody (ana), end-stage renal disease (esrd), esophagogastroduodenoscopy (egd), microscopic polyangiitis (mpa), myeloperoxidase (mpo)

## Abstract

Microscopic polyangiitis (MPA) is a rare, necrotizing vasculitis that primarily affects small-sized blood vessels, with a predilection for the upper and lower respiratory tract and the kidneys. It is commonly associated with the presence of perinuclear antineutrophil cytoplasmic antibodies (p-ANCA), often targeting myeloperoxidase (MPO), and typically presents with renal impairment, pulmonary symptoms, or systemic features such as fatigue and weight loss.

We report the case of a 76-year-old Caucasian woman who presented with anemia, dark stools, and acute kidney injury (AKI) but lacked significant pulmonary symptoms. Her evaluation revealed chronic sinusitis on imaging and pauci-immune necrotizing glomerulonephritis (GN) on kidney biopsy, with perinuclear antineutrophil cytoplasmic antibody (p-ANCA) positivity and limited crescent formation. Despite the absence of overt respiratory or constitutional symptoms, her diagnosis of MPA was confirmed, and she was initiated on rituximab therapy with plans for outpatient rheumatology and nephrology follow-up. This case highlights the importance of considering vasculitis in elderly patients presenting with unexplained renal dysfunction and subtle upper respiratory findings, even in the absence of classic systemic features.

## Introduction

Microscopic polyangiitis (MPA) is a rare, necrotizing vasculitis affecting small-sized arteries with a predilection for the upper and lower respiratory tracts and kidneys. It is more predominant in people of Asian descent, although it can affect other ethnicities as well [1]. Prevalence ranges from nine to 95 cases per one million persons [1]. Most patients with MPA present initially with nonspecific findings such as fever, malaise, myalgias, and arthralgias, and as a result, MPA may be misdiagnosed as infections, rheumatological abnormalities, or even early signs of cancer, which may lead to cumbersome testing [2]. Typical symptoms include rhinosinusitis, cough, hemoptysis, dyspnea, hematuria, proteinuria, and urine sediments with/without kidney impairment, although other cutaneous and neurological symptoms may also coexist [3]. Definitive diagnosis is tissue biopsy, which is guided by the site suspected to be involved in the disease process, for example, kidney, lung, or skin biopsy [4]. Treatment usually involves high-dose corticosteroids and a cytotoxic agent such as rituximab [5].

## Case presentation

This is a case of a 76-year-old woman who presented to the emergency department (ED) from her primary care physician’s (PCP) office due to abnormal laboratory results, notably a low hemoglobin (Hb) of 5.6 g/dL. Her past medical history included type 2 diabetes mellitus, hypertension, hyperlipidemia, coronary artery disease, congestive heart failure, and chronic kidney disease with a Kidney Disease Improving Global Outcomes (KDIGO) stage 2.

Furthermore, the patient had an endoscopic gastroduodenoscopy (EGD) three weeks preceding her ED visit for the evaluation of acute-on-chronic anemia due to a history of upper gastrointestinal (GI) bleeding. The results of the EGD showed Los Angeles (LA) grade D esophagitis (one or more mucosal breaks involving at least 75% of the esophageal circumference) with bleeding, gastritis, and a non-bleeding duodenal ulcer. Biopsies were taken, and clips were placed. She was admitted for the EGD findings due to her comorbidities, and during this admission, she admitted to taking copious amounts of nonsteroidal anti-inflammatory drugs (NSAIDs) for pain and reported several episodes of black tarry stools. She was started on intravenous (IV) pantoprazole 40 mg twice daily and later transitioned to oral pantoprazole 40 mg for three months in addition to sucralfate suspension 1,000 mg twice daily for two weeks. The patient also reported a history of recurrent sinus congestion and sinusitis requiring frequent antibiotics over a span of two years.

When she was seen at the ED for low Hb, the patient reported dyspnea on exertion, including with very minimal activity, and increasing lower extremity swelling, which originally prompted her visit to her PCP. On the review of systems, she reported weakness, sinus pressure without headaches, lethargy, and dark stools but denied blood in her urine, fever, chills, cough, or abdominal pain. Physical examination revealed a pale-appearing woman in no apparent distress with bilateral pitting edema up to the knees. She was afebrile, with normal blood pressure, and tachypneic with a respiratory rate of 20 breaths per minute, but no murmurs or tachycardia were noted. She was oriented to person, place, and time and had warm and well-perfused extremities. Pertinent laboratory results included a creatinine of 5.5 mg/dL, which was increased from a baseline of 1.0 mg/dL, and an estimated glomerular filtration rate (eGFR) of 8 mL/minute/1.73 m^2^, which was decreased from her baseline of >60 mL/minute/1.73 m^2^ (Table 1).

**Table 1:** Laboratory results on admission BUN, blood urea nitrogen; eGFR, estimated glomerular filtration rate

Parameter	Value	Reference range
Sodium	130	135-145 mmol/L
Potassium	4.4	3.5-5.0 mmol/L
BUN	103	6-20 mg/dL
Creatinine	5.5	0.5-1.0 mg/dL
eGFR	8	>60 mL/minute
Calcium	8.3	8.4-10.2 mg/dL
Phosphorus	3.6	2.5-5.0 mg/dL

Previous urinalysis performed three weeks prior to admission revealed blood in the urine, red blood cells (RBCs), and hyaline casts. Gastroenterology was consulted due to suspected bleeding ulcers being the etiology of her low Hb, and the patient underwent EGD the next day, which showed LA grade B reflux esophagitis (one ore more mucosal breaks >5 mm long that does not extend between the tops of two mucosal folds) with no bleeding; polypoidal tissue was also found in the lower third of the esophagus. Biopsies were taken. There were no esophageal ulcers found. Nephrology was also consulted in the ED due to acute-on-chronic kidney injury, and intravenous iron (Venofer 200 mg) and epoetin alfa were recommended.

Due to worsening renal function (urine output decreased from 65 mL/hour to 40 mL/hour), the patient was bladder-scanned. Bladder scan revealed no post-void residual volume, and daily furosemide 80 mg IV was initiated; however, the patient’s kidney function continued to decline despite management with furosemide. Subsequent urinalysis the next day was positive for blood and RBCs. The urine albumin/creatinine ratio was elevated to 1,129 mg/g from 129 mg/g three months prior (normal range: <30 mg/g), as was the urine protein/creatinine ratio at 2,596 mg/g. Creatinine worsened from 5.5 mg/dL on admission to 6.2 mg/dL on hospital day 9, and lower extremity edema up to the knees persisted despite intravenous diuretics. She also continued to have gross hematuria, progressive dyspnea with minimal exertion, progressive fatigue, and cough with sinus congestion but no epistaxis.

**Figure 1:**
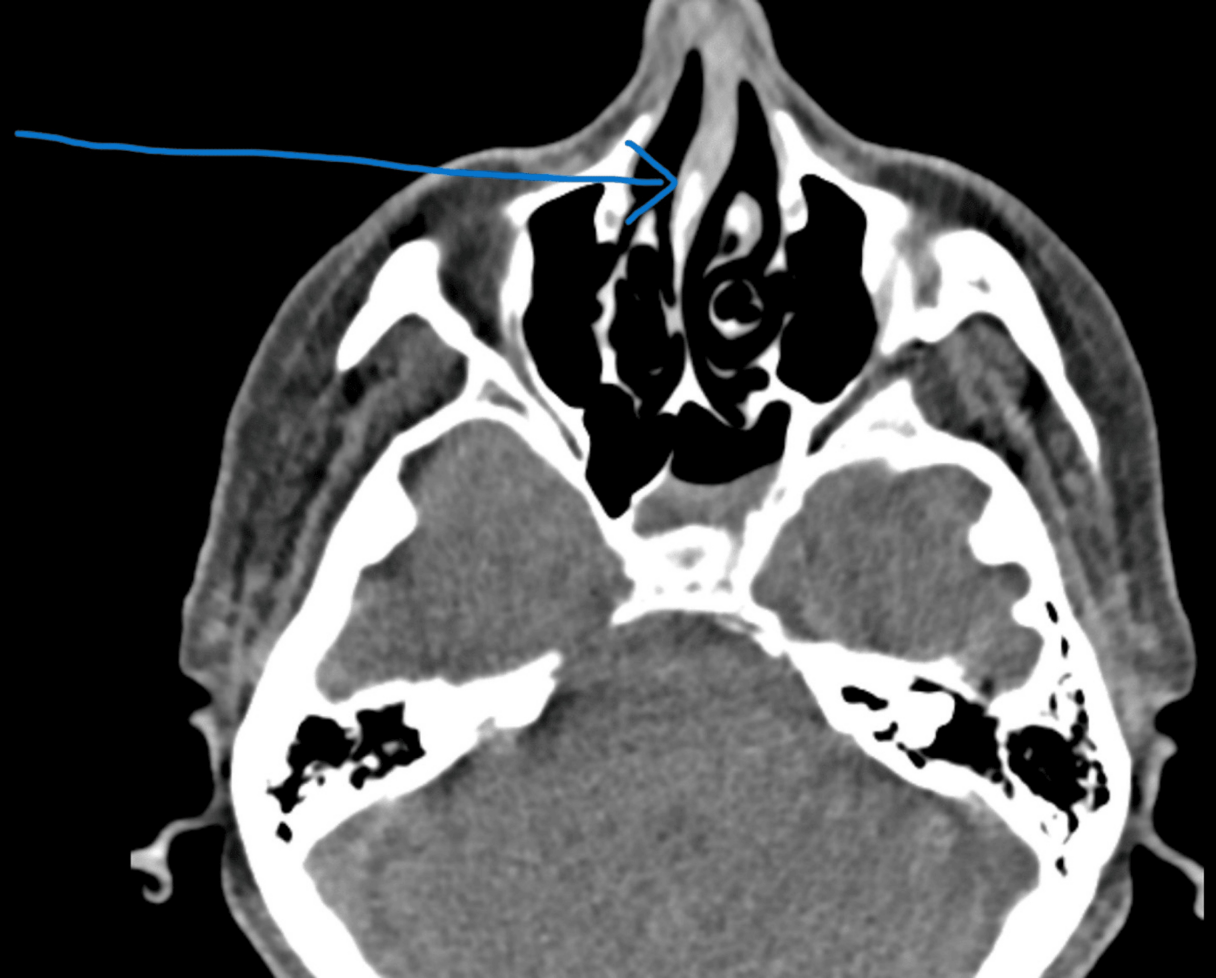
CT scan of the sinuses without contrast showing nasal septum deviation and chronic left sphenoidal sinusitis The arrow shows a deviated nasal septum

Due to her constellation of symptoms, it was suspected that she had an underlying rheumatological disorder, with vasculitis at the top of the differential. As a result, rheumatology was consulted. An antinuclear antibody (ANA) screen was ordered and was positive. Further testing also revealed a positive perinuclear antineutrophil cytoplasmic antibodies (p-ANCA), also known as myeloperoxidase (MPO)-ANCA, and a CT scan of the sinuses showed acute-on-chronic left sphenoidal sinusitis (Figure 1).

**Figure 2:**
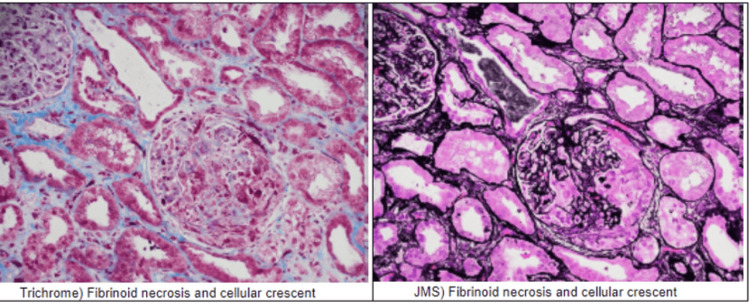
Kidney biopsy results Three glomeruli exhibit segmental glomerular tuft necrosis with associated cellular crescent formation. One fibrocellular crescent is also noted. There is moderate to severe interstitial fibrosis and associated tubular atrophy, estimated at 40%-50%. The trichrome stain, under light microscopy, shows tubules that exhibit conspicuous proteinaceous casts, but no atypical casts or crystals are seen. The JMS stain demonstrates similar findings, under direct immunofluorescence JMS: Jones’ Methenamine Silver

Kidney biopsy was performed and revealed focal pauci-immune necrotizing glomerulonephritis (GN), associated with p-ANCA, with 3/25 cellular crescents and 1/25 fibrocellular crescents (Figure 2).

The patient subsequently received pulse dose steroids (methylprednisolone 1,000 mg daily for two doses) and rituximab 375 mg/m^2^ once daily for two doses and was advised to follow up with rheumatology and nephrology following discharge from the hospital.

## Discussion

MPA is a primary systemic vasculitis characterized by the inflammation of the small blood vessels in almost any organ or tissue, although the kidneys and upper and lower respiratory tracts are most commonly affected [1]. It is also characterized by the presence of circulating antineutrophil cytoplasmic antibodies (ANCA) [2]. It is a subset of the primary systemic vasculitides that include Wegener’s granulomatosis (WG), Churg-Strauss syndrome (CSS), and renal-limited vasculitis [2]. MPA is more prevalent in people of Asian descent, but several studies have also found that Caucasians are two- to threefold more likely to be affected than other ethnicities [3].

MPA most commonly occurs in older adults, with an average age of onset of 50-60 years [6]. Patients may initially present with nonspecific symptoms such as fever, myalgia, fatigue, weight loss, and arthralgias, which may last days to weeks [2], but 65% of patients progress to have chronic symptoms over months to years that typically present as recurrent rhinosinusitis, cough, hemoptysis, hematuria, and proteinuria [4].

When the ear, nose, and throat are involved, 35% will present with nasal crusting, persistent rhinorrhea, purulent nasal discharge, and nasal ulcers [5]. Around 35%-55% of MPA patients will present with pulmonary manifestations, including hemoptysis and alveolar hemorrhage, infiltrates, pulmonary edema, and interstitial fibrosis [7].

The classic pulmonary manifestation is diffuse alveolar hemorrhage caused by pulmonary capillaritis [7]. Chest radiographs in these patients show patchy, bilateral airspace opacities, usually involving both the upper and lower lung fields, with ground-glass attenuations being the most common CT finding, although findings are variable [8]. Kidney involvement is by far the most significant clinical feature and frequently presents as proteinuria with up to 50% presenting with nephrotic range proteinuria (i.e., >3.5 g/24 hours), hematuria, and white blood cell (WBC) casts in urine [8]. Rapidly progressive glomerulonephritis is the typical presentation of MPA [9], and approximately 80% of patients with MPA develop renal complications that can range from asymptomatic urinary sediments to end-stage renal disease (ESRD) requiring renal replacement therapy [10]. ANCA plays a significant role in the pathogenesis of MPA through two modelled pathways [5].

First, neutrophils are activated by exposure to pro-inflammatory cytokines such as IL-1, which leads to the surface expression of MPO or proteinase-3 (PR3), followed by the adherence of these neutrophils to the endothelial surface of blood vessels and/or glomeruli. Second, neutrophils are activated either by interaction between surface MPO and p-ANCA or surface PR3 and anti-PR3 antibodies (also known as cytoplasmic ANCA or c-ANCA); neutrophils may also be activated through the binding of p-ANCA or c-ANCA directly to neutrophil Fc receptor [10].

It should be noted that the absence of kidney disease does not rule out or mitigate the severity of the disease process, as other organ systems such as the heart, GI tract, and central nervous system (CNS) may be involved and can be equally life-threatening. As seen in this case, the patient presented with recurrent anemia with upper endoscopy showing gastric ulcers, but the constellation of symptoms prompted further evaluation into the cause of hematuria, proteinuria, and sinus symptoms.

Differential diagnosis

MPA first manifests with nonspecific symptoms as mentioned above, and differentiating between ANCA-associated vasculitis and other systemic inflammatory diseases is often a challenge. As a result, MPA may be misdiagnosed or completely missed. Other common diseases with similar presentation to MPA may include anti-glomerular basement membrane (anti-GBM) antibody disease, drug-induced ANCA vasculitis, eosinophilic granulomatosis with polyangiitis, infections, malignancy, and other underlying glomerular diseases [11]. Although patients with anti-GBM disease will present with fever, hematuria, arthralgia, and a rapid decline in kidney function, renal biopsy would show a linear staining for anti-GBM antibodies [11].

In our case, a kidney biopsy helped rule out anti-GBM. Furthermore, the patient was not taking any medications such as minocycline, propylthiouracil, or hydralazine that have been associated with drug-induced ANCA vasculitis. However, causal associations between these drugs and ANCA-associated vasculitis are not well-established, and drug-induced ANCA-associated vasculitis is relatively uncommon, so the possibility of this complication should not preclude the use of these medications [12].

Eosinophilic granulomatosis with polyangiitis is almost indistinguishable from MPA in the absence of asthma and peripheral blood eosinophilia [13]. Patients with Churg-Strauss syndrome usually present with allergic rhinitis and have a positive history of atopy and peripheral eosinophilia, of which the latter two symptoms were conspicuously absent in our patient [13]. Patients with Churg-Strauss syndrome who receive inhaled glucocorticoids and omalizumab might experience worsening symptoms of rhinitis and atopy; such symptomatic exacerbation in a patient suspected to have simple atopic rhinitis or asthma might be indicative of Churg-Strauss syndrome [14]. Our patient was not on any medications for asthma, and she denied any history of asthma. The cutaneous manifestations in MPA include skin nodules, urticaria, and lower extremity purpura and ulcerations [15]. These skin manifestations may show some overlap with the cutaneous symptoms seen in polyarteritis nodosa (PAN); however, polyarteritis nodosa also presents with visceral microaneurysms that commonly manifest as abdominal pains, renal infarcts, and myocardial infarctions, a major point of differentiation between MPA and PAN [1]. PAN is typically ANCA-negative, meaning that it is negative for antibodies against both MPO and PR3. Given the absence of consistent cutaneous findings, subjective symptoms supporting a diagnosis of PAN, and corroborating imaging findings such as renal infarcts, PAN was unlikely in this patient.

Diagnosis and treatment

The diagnosis of MPA involves a good history and physical examination, laboratory testing for ANA and ANCA (including anti-MPO and anti-PR30), chest radiograph, and chest CT in patients suspected to have pulmonary symptoms. The definitive diagnosis is confirmed by the biopsy of the suspected organ involved (e.g., the kidney, lung, or skin) [11]. The severity of kidney biopsy findings usually parallels the severity of the clinical presentation, ranging from mild focal segmental glomerulonephritis (FSGN) in patients with near-normal kidney function with or without asymptomatic hematuria to a diffuse necrotizing and crescentic GN in patients with acute or acute-on-chronic kidney injury, as seen in our patient [16].

Some clinicians might decide to initiate treatment without biopsy in severely ill, ventilator-dependent patients, in whom obtaining an organ biopsy is considered high-risk and likely to be associated with poor outcomes.

Treatment initiation without preceding confirmatory biopsy may also be considered in those whose clinical presentation is highly consistent with pauci-immune vasculitis, in which the results of ANCA are unequivocal [16]. However, every attempt should be made to confirm the diagnosis by tissue biopsy before initiating treatment. Once diagnosis is established, treatment involves an initial induction phase, followed by a maintenance phase. Induction therapy usually consists of a glucocorticoid in combination with either rituximab or cyclophosphamide. After remission is achieved, rituximab is subsequently used as maintenance therapy for a duration of 12-24 months [17]. Azathioprine, methotrexate, and mycophenolate may also be used for maintenance therapy. Patients who continue to relapse on therapy, or who develop ESRD, will ultimately need a kidney transplant [18].

## Conclusions

MPA is a systemic vasculitis that commonly affects older adults and can affect any organ in the body. It typically presents in a nonspecific manner with symptoms including fatigue, myalgia, rhinosinusitis, hematuria, and kidney dysfunction. The diagnosis is based on a combination of clinical symptoms, laboratory tests, and imaging studies. A positive ANCA test supports the diagnosis, although it is not specific for MPA. The histological examination of tissue biopsy obtained from the affected organ, for example, the kidney, lungs, or skin, remains the most definitive method to establish a formal diagnosis.

Histopathological examination typically reveals vasculitis without any or very minimal immune deposits on light microscopy and immunofluorescence. Treatment usually involves an induction phase with glucocorticoids with either rituximab or cyclophosphamide, and this is followed by a maintenance phase with rituximab after remission is achieved. The prognosis is poor for those with kidney involvement who do not respond to initial treatment. Clinicians should be on the lookout for MPA because some patients may present with nonspecific symptoms such as fever, malaise, myalgias, and anorexia, and symptoms may last for weeks to months without any specific organ involvement. A high index of suspicion is recommended for patients with rapidly progressing glomerulonephritis, especially if these patients have concomitant ear, nose, and throat symptoms and pulmonary or skin involvement, even in the absence of classic systemic features.
